# Active Cloaking of a Non-Uniform Scatterer

**DOI:** 10.1038/s41598-020-58706-z

**Published:** 2020-02-06

**Authors:** Paris Ang, George V. Eleftheriades

**Affiliations:** University of Toronto, The Edward S. Rogers Sr. Department of Electrical and Computer Engineering, Toronto, M5S 2E4 Canada

**Keywords:** Electrical and electronic engineering, Applied physics

## Abstract

An object illuminated by an electromagnetic wave can be actively cloaked using a surface conformal array of radiating sources to cancel out scattering. This method is promising as elementary antennas can be used as sources while its active nature can surpass passivity-based performance limitations. While this technique has been conceptually extended to accommodate complex geometries, experimental validation past simple uniform scatterers is lacking. To address this scarcity, the design and experimental demonstration of a low-profile, active cloak capable of concealing a complex, metallic, polygonal target is presented. This cloak is constructed with commercially available monopoles and enclosed within a parallel-plate waveguide-based apparatus to approximate a quasi-2D environment. Performance is then assessed when the target is illuminated at either frontal or oblique incidence by a 1.2 GHz cylindrical wave. Overall, the cloak reduces the target’s scattering cross-section by an average of 7.2 dB at frontal incidence and 8.6 dB at oblique incidence. These results demonstrate the feasibility of this kind of active cloaking for more complex scatterers containing flat surfaces and edges. Further analysis shows that the cloak possesses a functional bandwidth of 14% and can be reconfigured for single frequency operation over 0.8–1.8 GHz.

## Introduction

Developments in artificial media, such as metamaterials, have given rise to new applications focused on advancing the ability to control electromagnetic waves. Electromagnetic cloaking comprises one such area and focuses on preventing an object from scattering impinging electromagnetic waves. This is achieved by either redirecting an incident wavefront around an object^1–4^ or by altering the properties of a target to reduce or cancel out natural scattering^5–7^. Although much research focuses on managing microwaves, cloaks at other bands^8,9^ along with thermal^10^, acoustic^11^, and temporal^12^ devices have been proposed. Presently, the majority of research into cloaking has focused on passive techniques. While elegant and operationally simpler, passivity imposes fundamental design and performance limitations. These often require compromises on: operational bandwidth, directionality, polarization, along with the permissible size, geometry, and material of the cloak and target^13,14^.

While more complex and less popular, active techniques provide a potential means of relaxing or mitigating passivity based constraints. One avenue of development is to use metasurfaces^15,16^ and metamaterials^17,18^ augmented with active components or networks. The resultant devices function similarly to passive cloaks but exhibit expanded capabilities^19^, reconfigurability^20,21^, or unidirectional reflectivity^22,23^. This present work focuses on an alternative concept, analogous to acoustic noise cancellation, where an active mechanism is employed to directly manage scattering^24,25^. Here, an illuminated object is surrounded by a surface conformal array of radiating elements which are configured to radiate a field discontinuity that cancels out scattering. This method was first experimentally demonstrated on a quasi-2D, metallic, circular cylinder with loop antenna elements in 2013^26^. The ability to employ elementary antennas represents a major advantage, greatly expanding operational flexibility and simplifying design. Recent, ongoing research includes efforts to expand this technique’s practicality; development of active cloaks for irregularly shaped 2D targets^27^, and versatility; employing thin, conformal patch-based elements to reduce the cloak’s profile^28^. Although successful, these designs have only been conceptually assessed within electromagnetic simulations.

This paper aims to continue this development track^29^ by presenting the experimental demonstration of a low-profile cloak, constructed with commercial antennas, designed to reduce the scattering of a 5-sided metallic polygonal target. Unlike the circular cylinders used in previous experiments, a polygonal target is more representative of real-world geometries and contains a variety of flat surfaces and edges. Furthermore, its irregular geometry causes it to scatter non-uniformly making the scattered fields angle dependent. The cloak design also features the employment of commercial monopole based elements which are found to be simpler, lower profile, and better performing than previous loop^26^ and quarter-wave patch^28^ designs. To replicate a quasi-2D free-space environment, the resultant cloak/target assembly is enclosed within an absorber-lined parallel-plate waveguide and illuminated at either a frontal or oblique incidence by a 1.2 GHz cylindrical wave to assess performance. This is then followed with an initial conceptual and experimental examination of the cloak’s multi-frequency capabilities.

## Results

### Active electromagnetic cloaking

**Figure 1:**
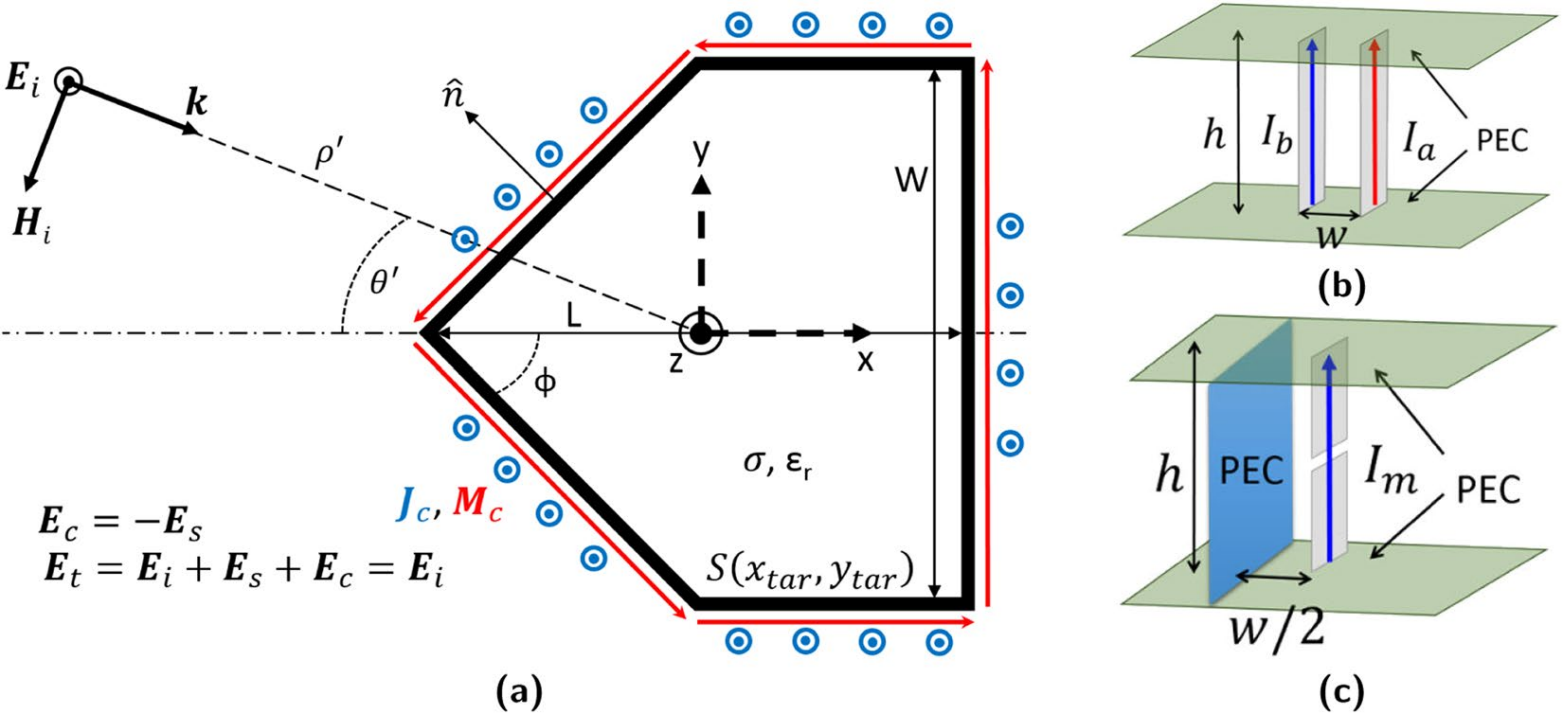
(**a**) Cloaking a five-sided polygonal target (**b**) Hybrid and (**c**) Simplified equivalence sources^32^.

 Figure 1a shows the conceptual layout of a cloak designed to hide a 5-sided polygonal target. Here, an arbitrary source positioned at ${\boldsymbol{\rho }}^{\prime} (\rho ^{\prime} ,\Theta ^{\prime} )$, where $\Theta ^{\prime} =\pi -\theta ^{\prime}$, radiates an incident electromagnetic (EM) wave with electric and magnetic components (**E**_**i**_, **H**_**i**_). This wave impinges upon the target at an incident angle of $\theta ^{\prime}$ and subsequently scatters, producing secondary scattered fields inside (**E**_**int**_, **H**_**int**_) and outside (**E**_**s**_, **H**_**s**_) the target. Along the target’s surface, ${\boldsymbol{\rho }}\left({x}_{tar},{y}_{tar}\right)$, these fields are related by:

1 $$\widehat{{\bf{n}}}\times {{\bf{E}}}_{{\bf{i}}}+\widehat{{\bf{n}}}\times {{\bf{E}}}_{{\bf{s}}}=\widehat{{\bf{n}}}\times {{\bf{E}}}_{{\bf{int}}}$$

2 $$\widehat{{\bf{n}}}\times {{\bf{H}}}_{{\bf{i}}}+\widehat{{\bf{n}}}\times {{\bf{H}}}_{{\bf{s}}}=\widehat{{\bf{n}}}\times {{\bf{H}}}_{{\bf{int}}}$$

with $\widehat{{\bf{n}}}$ representing the outward facing normal. The Huygens’ equivalence principle is then applied to these scattered fields, allowing them to be represented as originating from surface-impressed, radiating sources on the target^30^. These are expressed by the magnetic (**M**_**s**_) and electric (**J**_**s**_) surface current densities^25,29^:

3 $${{\bf{M}}}_{{\bf{s}}}=-\,\widehat{{\bf{n}}}\times \left({{\bf{E}}}_{{\bf{s}}}-{{\bf{E}}}_{{\bf{int}}}\right)$$

4 $${{\bf{J}}}_{{\bf{s}}}=\widehat{{\bf{n}}}\times \left({{\bf{H}}}_{{\bf{s}}}-{{\bf{H}}}_{{\bf{int}}}\right)$$

which if implemented, will radiate the same scattered field distribution in the absence of the original incident wave. Rearranging and substituting (1) and (2) reduces these to^25^:

5 $${{\bf{M}}}_{{\bf{s}}}=\widehat{{\bf{n}}}\times {{\bf{E}}}_{{\bf{i}}}$$

6 $${{\bf{J}}}_{{\bf{s}}}=-\,\widehat{{\bf{n}}}\times {{\bf{H}}}_{{\bf{i}}}$$

meaning the scattered fields and representative source currents can be deduced with only knowledge of the incident fields. An active cloak is then created by impressing these sources into the target with a 180° phase offset (**M**_**c**_ = −**M**_**s**_, **J**_**c**_ = −**J**_**s**_). The resultant phase-inverted fields (**E**_**c**_, **H**_**c**_) radiated by these sources then cancel out their natural scattered counterparts when the source-equipped target is re-illuminated by the original incident wave.

### Cloaking elements

The cloaking solution can be physically implemented with an array of *n* input weighted radiating elements positioned around the target’s perimeter. Weights for each element are calculated by first discretizing the continuous magnetic and electric surface currents from (5) and (6) into *n* individual dipole moments:

7 $${{\boldsymbol{\rho }}}_{m}={{\bf{M}}}_{{\bf{c}}}({x}_{n},{y}_{n})hl$$

8 $${{\boldsymbol{\rho }}}_{e}={{\bf{J}}}_{{\bf{c}}}({x}_{n},{y}_{n})lh$$

where *l* is the spacing between element centers, *h* is the target’s height, while **M**_**c**_(*x*_*n*_, *y*_*n*_) and **J**_**c**_(*x*_*n*_, *y*_*n*_) are the magnetic and electric currents at the location of a specific element. Any conventional antenna capable of generating these moments can then be employed as an element. For example, magnetic moments can be generated from loop antennas (of area *A*), with an input current of: ${I}_{m}={{\boldsymbol{\rho }}}_{m}/\left(j\omega \mu A\right)$^31^, and patch antennas^28^. Similarly, a wire dipole antenna with input: *I*_*e*_ = ***ρ***_*e*_/*h* produces an electric moment.

An alternative to separate electric and magnetic arrays is to simultaneously generate both electric/magnetic moments using hybrid elements. One example is the “equivalence source” in Fig. 1b^32^, composed of a dipole or monopole antenna pair, each individually current fed with:

9 $${I}_{a}=0.5{I}_{e}+{I}_{m}$$

10 $${I}_{b}=0.5{I}_{e}-{I}_{m}$$

The 0.5*I*_*e*_ currents in each antenna forms an even mode which approximates an electric dipole with input *I*_*e*_. Conversely, the odd mode arising from the opposing ±*I*_*m*_ currents approximates a current loop with input *I*_*m*_.

In the presence of a conductive target, the electrical even mode is shorted out (**J**_**c**_ = 0, *I*_*b*_ = −*I*_*a*_) and the source can be simplified to a single antenna (Fig. 1c). The image effect mirrors the antenna’s electric current across the conductive plane, creating a virtual current loop and the required magnetic odd mode^29,32^.

### Experimental setup

**Figure 2:**
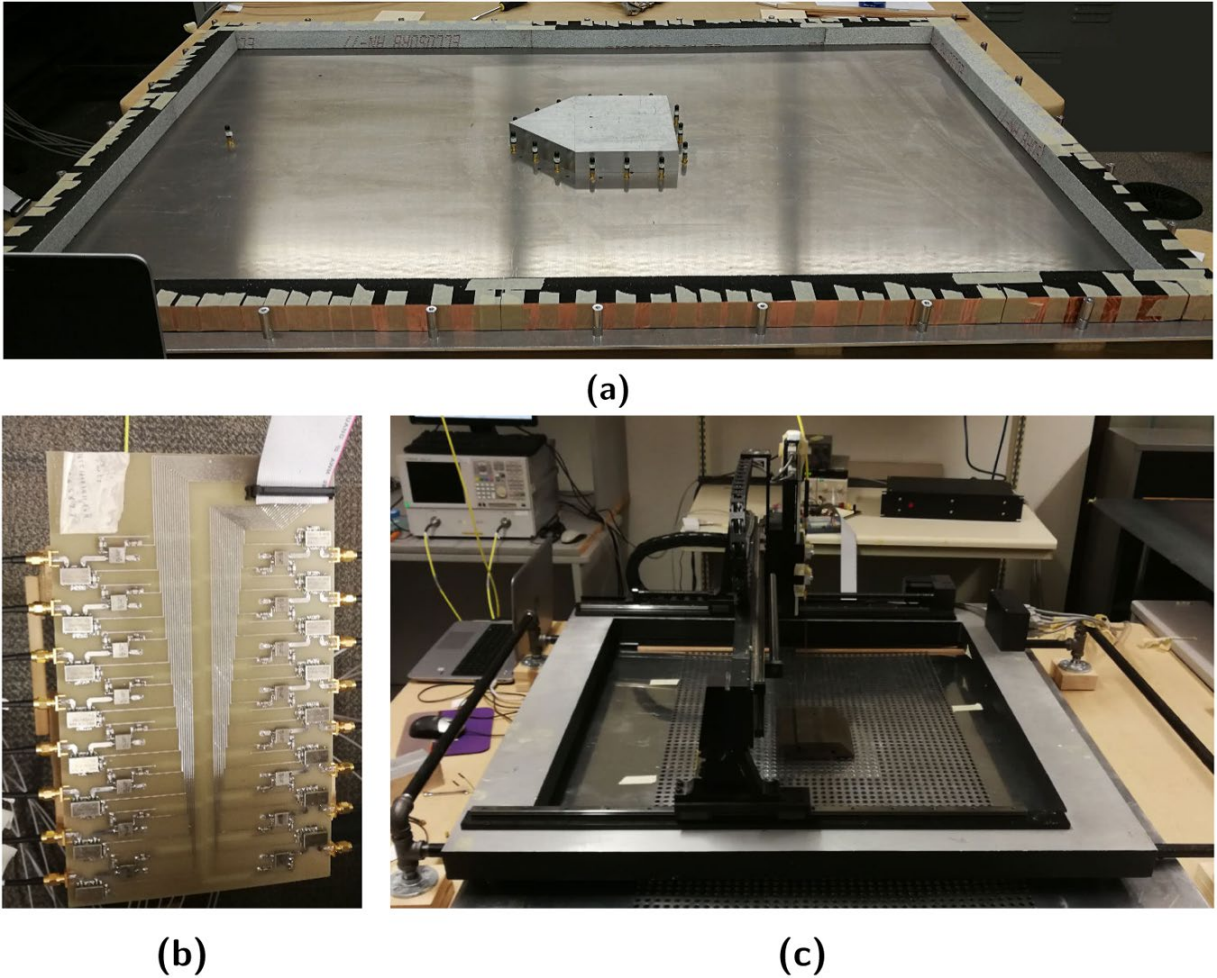
Experimental setup (**a**) Open waveguide (**b**) Weighting controller (**c**) Closed waveguide & translator stage.

The experimental setup pictured in Fig. 2a physically represents the layout in Fig. 1b. Here, the cloak array, source antenna, and aluminum polygonal target (*L* = 272.73 mm, *W* = 218.18 mm, and *ϕ* = 45°) are affixed to the aluminum baseplate (1626 × 1094 mm) of a parallel-plate waveguide. To suppress non-TEM modes and impose quasi-2D conditions, the waveguide plate spacing and target height are selected to be *h* = 38 mm; less than half a wavelength at the operational frequency of 1.2 GHz. The waveguide’s perimeter is then lined with absorbers to replicate a free-space region. It should be noted that the design of this experiment has been partially documented within a previous conference publication^29^.

Due to the target’s conductive nature, the cloak utilized the purely-magnetic reduced element design from Fig. 1c. These took the form of 2.4 GHz commercial monopoles mounted *w/*2 = 14 mm from the target’s surface and operated off-resonance to mitigate antenna-based scattering. A total of *n* = 15 cloak elements were used, distributed with three, equally spaced, antennas per face. The resultant inter-elemental spacing on each face was less than half-wavelength, ensuring that the array possessed sufficient resolution to reproduce the scattered field. Employing commercial monopoles was advantageous as they were inexpensive, performed consistently, and could be adapted to accommodate various configurations. Furthermore, these elements proved to be more compact and versatile than their patch^28^ and loop^27^ based predecessors.

**Figure 3:**
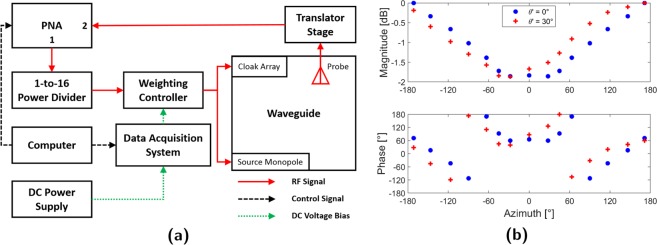
(**a**) Apparatus system diagram (**b**) Required magnetic currents.

A single source monopole would then be mounted in one of two baseplate holes, drilled $\rho ^{\prime} =600$ mm from the target’s center, to illuminate the target with a cylindrical wave at either $\theta ^{\prime} ={0}^{\circ }$ (frontal) or $\theta ^{\prime} =3{0}^{\circ }$ (oblique) incidence. Coupled with the target’s polygonal geometry, these incidence cases would represent scattering off acute and oblique edges along with flat surfaces. Feeding and weighting of both cloak and source antennas was facilitated by the 16-channel controller in Fig. 2b^32^. Each channel was equipped with a voltage-controlled attenuator and phase shifter pair to allow for individual control of each input. Fig. 2c shows the closed waveguide where an 80 × 40 array of 10 mm diameter perforations, spaced 15 mm apart, have been punched into the top plate. The fields within could then be mapped by an automated translator stage which inserts a coaxial probe into each hole and measures controller input referenced *S*_21_. Fig. 3a presents the full apparatus system diagram.

### Element weighting

Within the waveguide’s quasi-2D environment, the source monopole emits a z-polarized (TE) cylindrical wave characterized by E-field: ${{\bf{E}}}_{{\bf{i}}}={E}_{0}{H}_{0}^{\left(2\right)}(k\left|{\boldsymbol{\rho }}-{\boldsymbol{\rho }}^{\prime} \right|)\widehat{{\bf{z}}}$. ${H}_{0}^{\left(2\right)}$ represents the Hankel function of the second kind, *k* the wavenumber, ${\boldsymbol{\rho }}\left(\rho ,\theta \right)$ the location of an observation point, and ${\boldsymbol{\rho }}^{\prime} (\rho ^{\prime} ,\Theta ^{\prime} )$ the source location^29^. This field distribution can then be substituted into (5) and (6) to obtain the required cloaking source currents.

A conductive target eliminates internal electric or time-varying magnetic fields (**E**_**int**_ = **H**_**int**_ = 0) and shorts out electric sources (**J**_**c**_ = 0). As such, the experimental cloak now only needs to approximate a magnetic surface current:

11 $${{\bf{M}}}_{{\bf{c}}}=-{E}_{0}{H}_{0}^{(2)}(k|{\boldsymbol{\rho }}({x}_{tar},{y}_{tar})-{\boldsymbol{\rho }}{\rm{^{\prime} }}|)[\hat{{\bf{n}}}\times \hat{{\bf{z}}}]$$

where ***ρ***(*x*_*tar*_, *y*_*tar*_) is a vector representing an observation point on the target’s surface.

In contrast to a circular target, the polygonal geometry results in a discontinuous **M**_**c**_ distribution. Furthermore, the use of a cylindrical incident wave necessitates an analytical solution due to the presence of a Hankel function. As such, individual **M**_**c**_ values are obtained by analytically evaluating (11) at the location of each element. These are plotted in (Fig. 3b) for the frontal $\theta ^{\prime} ={0}^{\circ }$ and oblique $\theta ^{\prime} =3{0}^{\circ }$ incidence cases. For ease of interpretation, the resultant **M**_**c**_ values are azimuthally (*θ*) referenced to the frame in Fig. 1a. In actuality, the radial position of each element (*ρ*) also varies due to the non-circular geometry. Although only two incident angle cases are analyzed experimentally, full wave simulations verifying cloak operation at $\theta ^{\prime} ={0}^{\circ }$, 30°, 150°, and 180° incidence as well as against multiple sources are detailed within the Supplementary Information.

### E-field measurements: 0° incidence

To visually confirm cloak effectiveness, four normalized real, E-field plots are presented for each incident case detailing: the total field when the cloak is off (OFF), the field scattered by the uncloaked target (SC), the field generated by the cloak array (CO), and the total field with the cloak active (ON). Normalized real values are ideal for field visualization as they provide increased contrast and account for both magnitude and phase variations. Locations of interest are expressed in terms of coordinates (X, Y) referenced to the lower left measurement start point.

**Figure 4:**
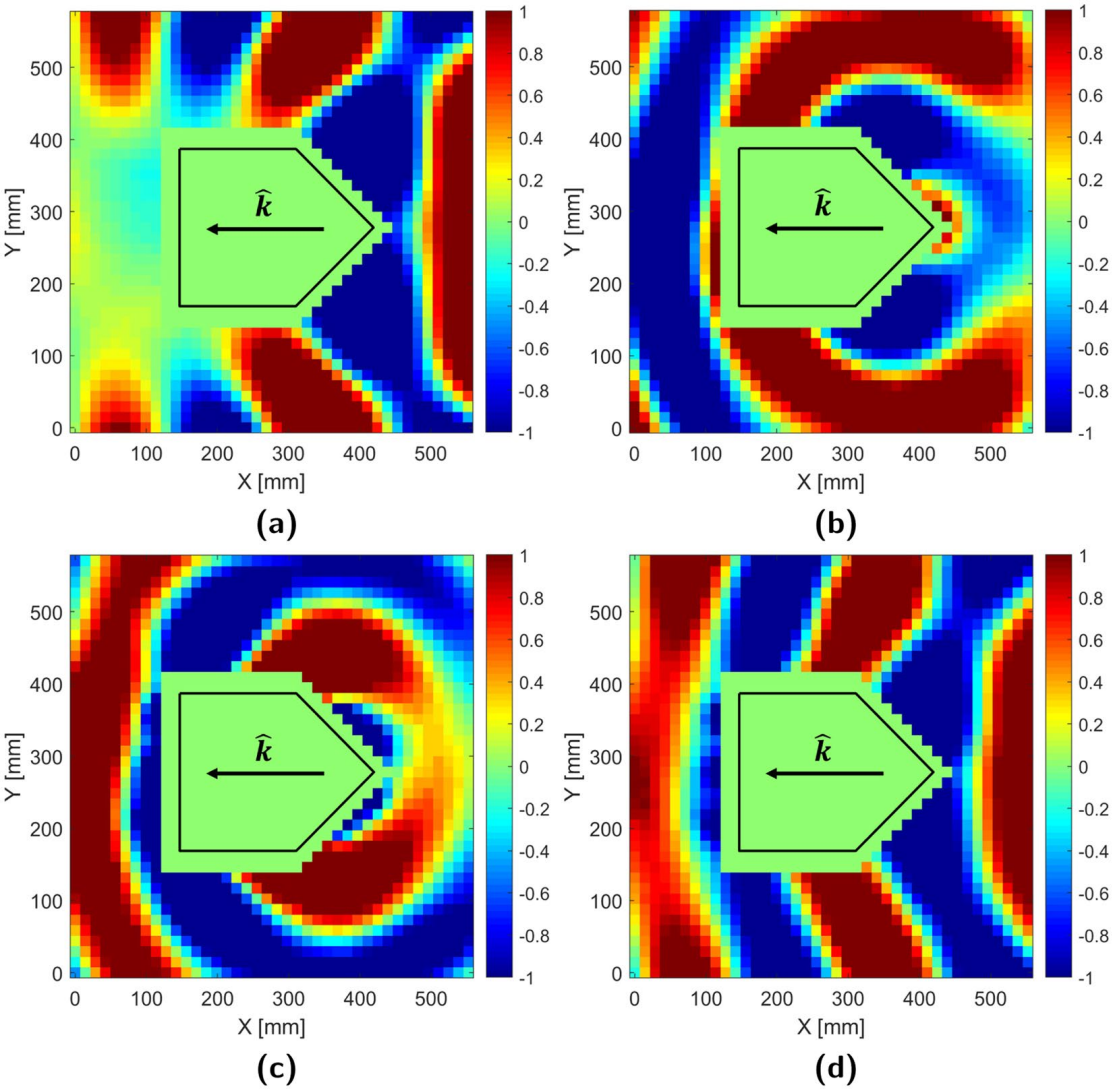
Normalized real E-field: $\theta ^{\prime} ={0}^{\circ }$ incidence (**a**) Total OFF (**b**) Scattered (SC) (**c**) Cloak (CO) (**d**) Total ON.

The OFF case measurements, plotted in Fig. 4a, show the total field distribution that occurs when an uncloaked target scatters an impinging electromagnetic wave. It can be seen that at $\theta ^{\prime} ={0}^{\circ }$ incidence, the cylindrical wave generated by the source directly impinges on the forward facing edge of the target (439 mm, 292.5 mm) and scatters off to the front and sides. These secondary scattered fields interfere with the original incident field, giving rise to wavefront distortion around the source-facing tip (>439 mm, 292.5 mm). The target also physically occludes the wave, causing the formation of a nearly field-free shadow region (<127.4 mm, 102.4–438.8 mm).

Subtracting the incident field distribution, taken with only the source in an empty waveguide, from the previous OFF field distribution isolates the aforementioned secondary scattered (SC) field. This is plotted in Fig. 4b and resembles an unbalanced cylindrical wave emanating from the forward facing point of the target. With proper configuration, the cloak array radiates the cloak (CO) field distribution in Fig. 4c; a phase-inverted copy of the SC field.

When added to the OFF distribution, the cloak field cancels out the scattered field resulting in the ON distribution in Fig. 4d. Here, it can be seen that frontal and side scattering at the source-facing tip (>439 mm, 292.5 mm) is suppressed resulting in a more cylindrical wavefront geometry. Furthermore, the shadow region behind the target has been largely filled in. Minor residual rippling, due to experimental apparatus and measurement imperfections, are observed near the target’s sides (439 mm, 482.6 mm) and rear (0–394.9 mm, 87.75–204.8 mm & 321.8–438.8 mm).

### E-field measurements: 30° incidence

**Figure 5:**
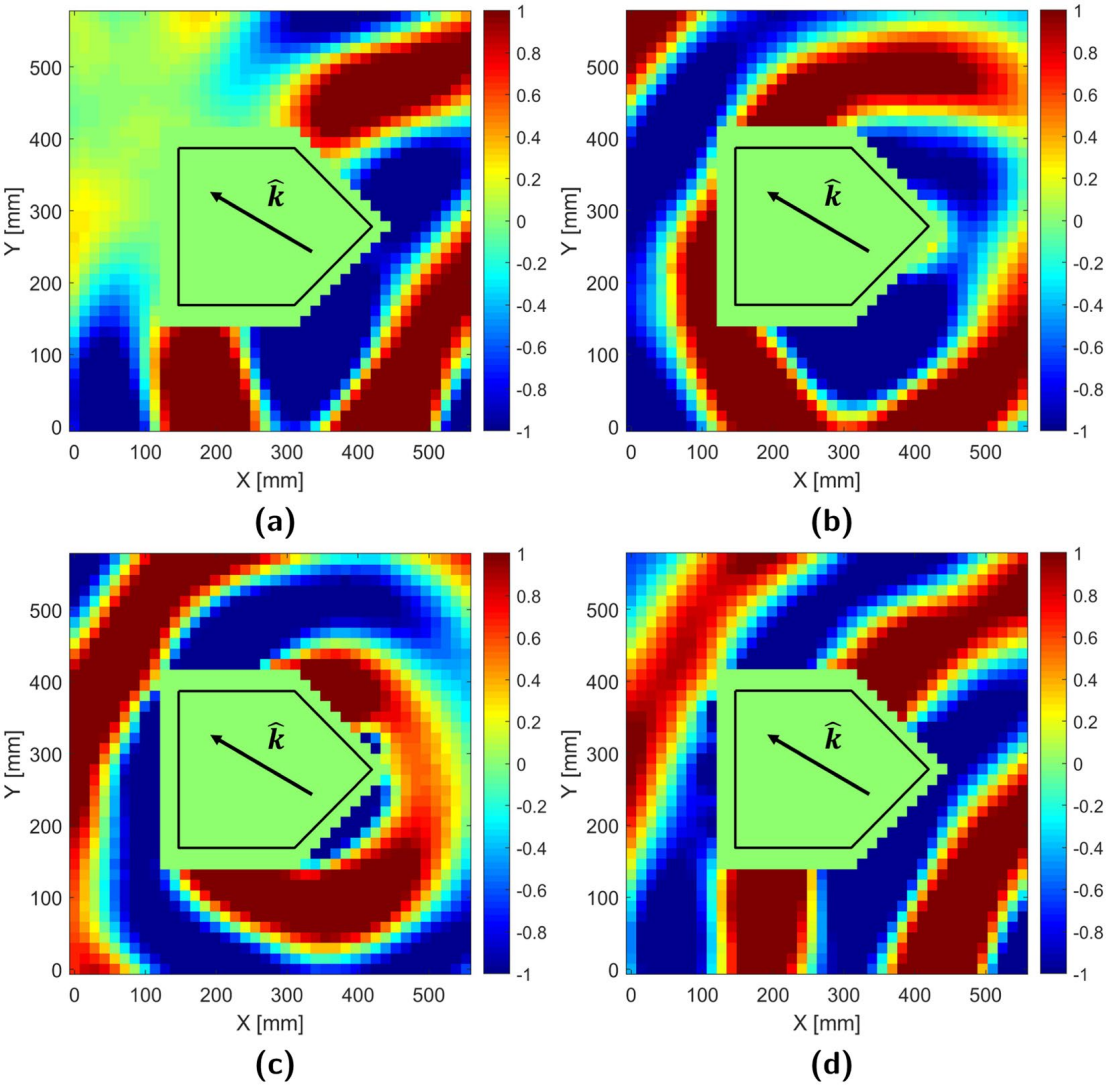
Normalized real E-field: $\theta ^{\prime} =3{0}^{\circ }$ incidence (**a**) Total OFF (**b**) Scattered (SC) (**c**) Cloak (CO) (**d**) Total ON.

 Figure 5a (OFF) shows that at $\theta ^{\prime} =3{0}^{\circ }$ incidence, the incident wave illuminates a wider target cross-section resulting in a larger shadow region. Additionally, the distortion in the frontal wavefront is asymmetric as the wavefront directly impinges on an angled flat surface (396.5 mm, 219.4 mm) rather than a symmetric edge.

Although the scattered (SC) field in Fig. 5b is still cylindrical, its orientation has been shifted along the new angle of incidence. As implied by the OFF case, the pattern is asymmetric due to disproportionate frontal specular reflection. Regardless, the cloak array (CO) successfully replicates and inverts the SC distribution (Fig. 5c).

ON case measurements (Fig. 5d) again indicate that much of the incident field has been successfully restored by the cloak. Some wave distortion is noted at the frontal sides of the target, (240.7–368.2 mm, 0 mm) and (396.5–538.1 mm, 424.1–555.8 mm) along with a region of reduced power in the former shadow region (0–184.1 mm, 380.3–541.1 mm).

### Bistatic radar cross-section

**Figure 6:**
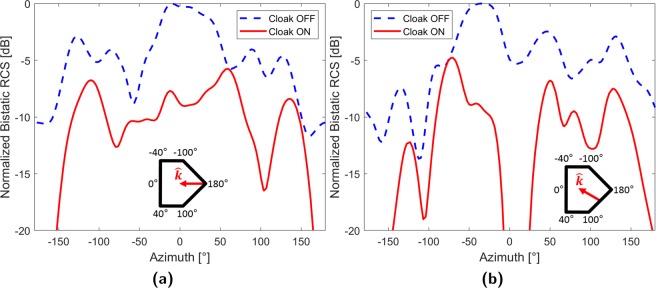
Bistatic radar cross-section patterns (**a**) $\theta ^{\prime} ={0}^{\circ }$ (**b**) $\theta ^{\prime} =3{0}^{\circ }$.

Bistatic radar cross-section (RCS) patterns provide a convenient means to quantify the scattering of an object. Within a 2D environment, bistatic RCS is defined azimuthally as $\sigma (\theta )={{\rm{lim}}}_{\rho \to \infty }2\pi \rho \frac{{\left|{{\bf{E}}}_{{\bf{s}}}\right|}^{2}}{{\left|{{\bf{E}}}_{{\bf{i}}}\right|}^{2}}$ where **E**_**s**_ and **E**_**i**_ are far-field measurements. As such, the near-field measurements obtained from the waveguide must first be extrapolated into the far-field. This is performed by treating the scattered field as a point source^26^ and applying a cylindrical harmonic expansion, originally devised for near-field antenna ranges, to obtain the far-field^33^ and determine the bistatic RCS:

12 $$\sigma (\theta )=\left|\mathop{\sum }\limits_{n=-\infty }^{\infty }{a}_{n}\left(\theta \right)\exp (jn\theta )\right|$$

with scattering coefficient:

13 $${a}_{n}\left(\theta \right)={\int }_{0}^{2\pi }\frac{{{\bf{E}}}_{{\bf{s}}}\left(\theta \right)\exp (-jn\theta )}{2\pi {H}_{n}^{(2)}\left(kb\right){H}_{n}^{(2)}(k\rho ^{\prime} )}\,d\theta $$

where ${{\bf{E}}}_{{\bf{s}}}\left(\theta \right)$ is calculated from field measurements sampled along a *b* = 270.94 mm radial track. The resultant RCS patterns are plotted in Fig. 6 and azimuthally referenced to the frame in Fig. 1.

 Figure 6a indicates that scattering suppression is achieved over the entire azimuth at $\theta ^{\prime} ={0}^{\circ }$ incidence. Additionally, the average suppression is determined to be 7.2 dB with a maximum of 36.3 dB at 173. 8° near the forward facing edge of the target. Conversely, suppression minima occur near the ±40° and ±100° edges with the global minimum of 0.1 dB encountered at 60. 1°. Due to the high amount of suppression at the directly illuminated edge, uniform azimuthal suppression, and low cloak OFF scattering at edges, it is unlikely that edge effects significantly impact overall cloak performance.

 Fig. 6b indicates that universal suppression is similarly achieved at $\theta ^{\prime} =3{0}^{\circ }$ incidence with an average of 8.6 dB. As with the $\theta ^{\prime} ={0}^{\circ }$ case, high suppression occurs near the 180° edge (36.3 dB at 173. 8°) with local minima manifesting near the other edges and a global minimum of 0.04 dB at −76°. Lastly, a region of high suppression is observed between −20° and 35° containing the global maximum of 37.8 dB (18. 1°). Supplementary Information also details full wave simulated patterns for $\theta ^{\prime} ={0}^{\circ }$, 30°, 150°, and 180° incidence as well as cloak performance against more than one incident source.

### Multiple frequency operation

To date, research in this method of active cloaking has focused solely on operation at a single design frequency. While still useful, the practical applicability of such a narrowband device is limited. As one of the main aims of this research is to advance the versatility of this technique, a conceptual exploration of multi-frequency cloaking is both appropriate and relevant. From a theoretical perspective, the frequency range of this active cloak is unconstrained. As long as the cloak is capable of generating the required **M**_*c*_ and **J**_*c*_ over the desired frequencies, the target will be successfully hidden. Instead, multi-frequency capabilities are primarily design and environmentally dependent.

The experimental cloak demonstrated in this paper serves to illustrate this point. As it was never intended for multi-frequency operation, the capabilities of the controller and sidewall absorbers severely limit the frequency range of the apparatus. Additionally, each channel in the cloak controller is only capable of enforcing a specific phase and amplitude at a single frequency. Regardless, this prototype remains useful in validating basic multi-frequency scenarios which can be built upon in future experimentation.

The first focus of this section is to assess the cloak’s ability to “frequency hop” or be reconfigured to operate at another single frequency. This is then followed by determining the cloak’s bandwidth when it is weighted at 1.2 GHz. As the frequency response of a real-world incident wave may be inconsistent and time varying, the utility of a single weight, wideband cloak may be limited. Instead, future designs may be versatile enough to enforce multiple weights at different frequencies, simultaneously generating multiple narrowband responses. These can be combined allowing the cloak to tailor its cancellation field for operation at multiple individual frequencies and/or over a frequency range. In this context, assessing the bandwidth of single weights would be useful for evaluating resolution requirements and interference between responses. As the geometry of the incident wave is largely frequency invariant, only a single ($\theta ^{\prime} ={0}^{\circ }$) incidence case needs to be considered.

#### Frequency hopping

**Figure 7:**
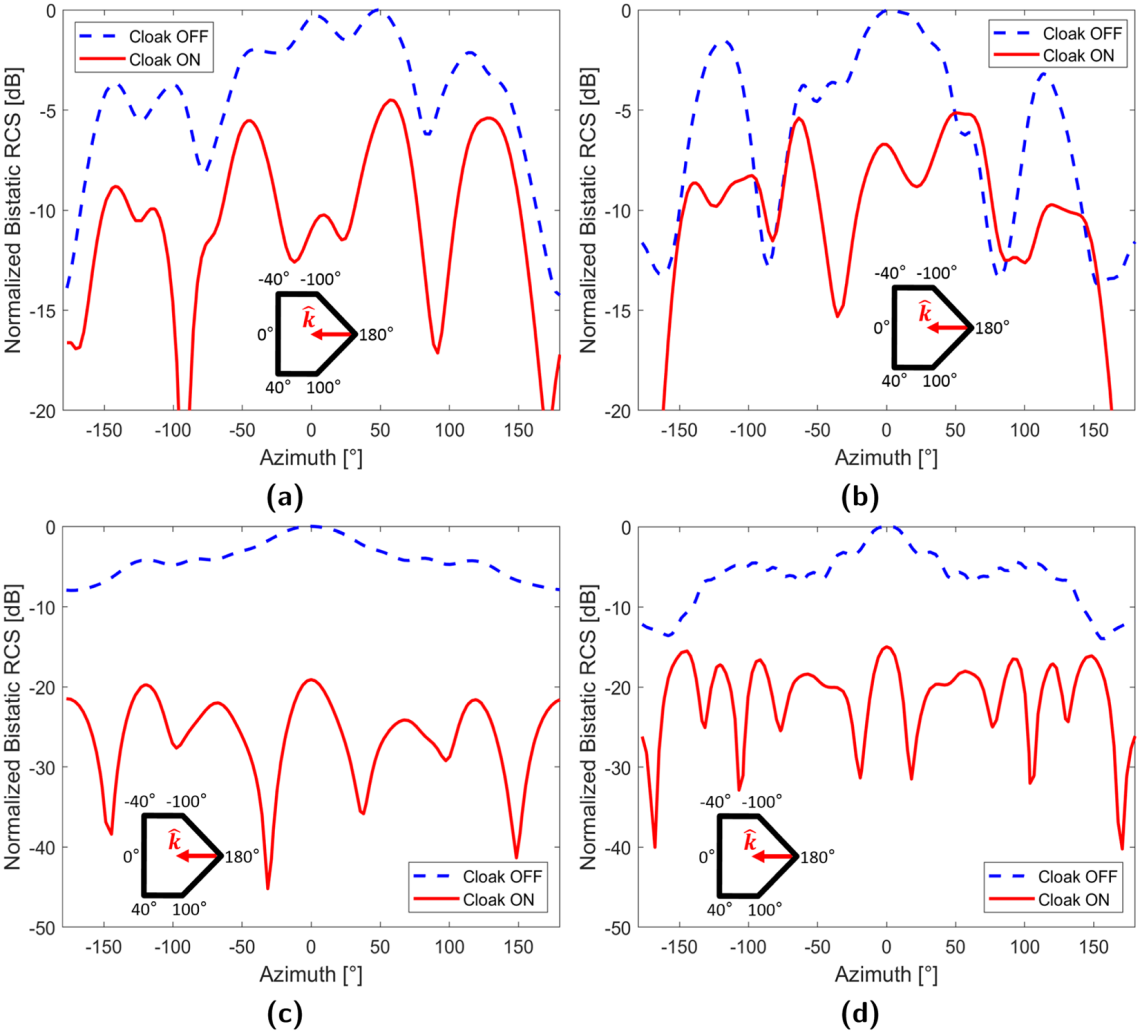
Frequency hopping (**a**) 1.1 GHz exp. (**b**) 1.3 GHz exp. (**c**) 0.8 GHz sim. (**d**) 1.8 GHz sim.

 Figure 7a shows the experimental bistatic RCS pattern obtained when the cloak is configured to operate at 1.1 GHz ($\theta ^{\prime} ={0}^{\circ }$). While cloaking is achieved over the entire azimuth, average suppression slightly decreases to 6.4 dB. This reduction is attributed to degraded absorber attenuation at lower frequencies causing excess sidewall scattering; manifesting as cloak ON maximas at ±50°. Conversely, the limitations of the controller prevents consistent and reliable element weighting at 1.3 GHz. The resultant implications can be seen in Fig. 7b where the cloak loses functionality around −89°, 73°, and 149° lowering average suppression to 5.1 dB.

**Figure 8:**
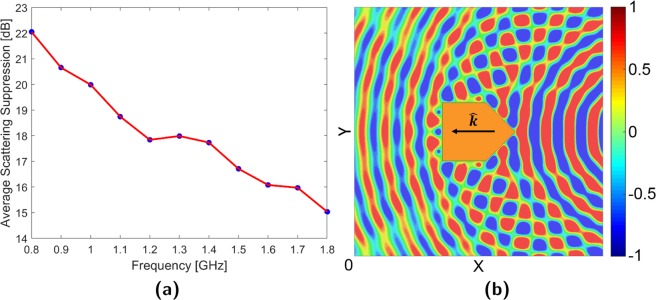
Frequency hopping simulations (**a**) Average scattering suppression (**b**) 3.5 GHz E-field.

To compensate for the limitations of the experimental apparatus, the parallel-plate apparatus was remodeled within a full-wave electromagnetic simulator. Although dimensionally identical to its experimental counterpart, the simulated waveguide was surrounded by perfectly matched layer (PML) boundary conditions rather than the experimental foam absorber. This eliminated sidewall scattering as the PML was perfectly absorbing over all frequencies. The removal of frequency based effects allowed the cloak to be reconfigured for single frequency operation across the range of 0.8–1.8 GHz. Bistatic RCS patterns at the sweep extremities were plotted in Fig. 7c,d along with the average suppression across this range in Fig. 8a. These indicated that scattering suppression consistently decreased with increasing frequency.

One explanation for this trend is the increase in resonant scattering from the cloak array as the operating frequency of the elements (2.4 GHz) is approached. If this additional scattered field component is not compensated for in array weighting, the field generated from the cloak will continue to cancel out only scattering by the target’s structure. Unfortunately, characterizing resonant scattering can be complex and may require computational solutions. At even higher frequencies, the performance of the cloak may be degraded by array aliasing and current phase inversion. Array aliasing occurs when the spacing of the cloak elements becomes larger than half the operational wavelength, preventing the array from accurately radiating a matching cloak field. This effect can be seen in Fig. 8b where, at 3.5 GHz, the E-field to the sides of the target becomes highly distorted. In contrast, the cloak elements at the back of the target align with the incident wave’s constant phase direction and possess enough resolution to partially restore the wavefront. Furthermore, if the electrical size of the elements becomes too large, the currents within may phase invert and cancel out. Both high frequency effects can be managed by keeping element length (for dipoles and monopoles) and spacing less than half-wavelength at the highest expected operating frequency.

#### Single weight bandwidth

**Figure 9:**
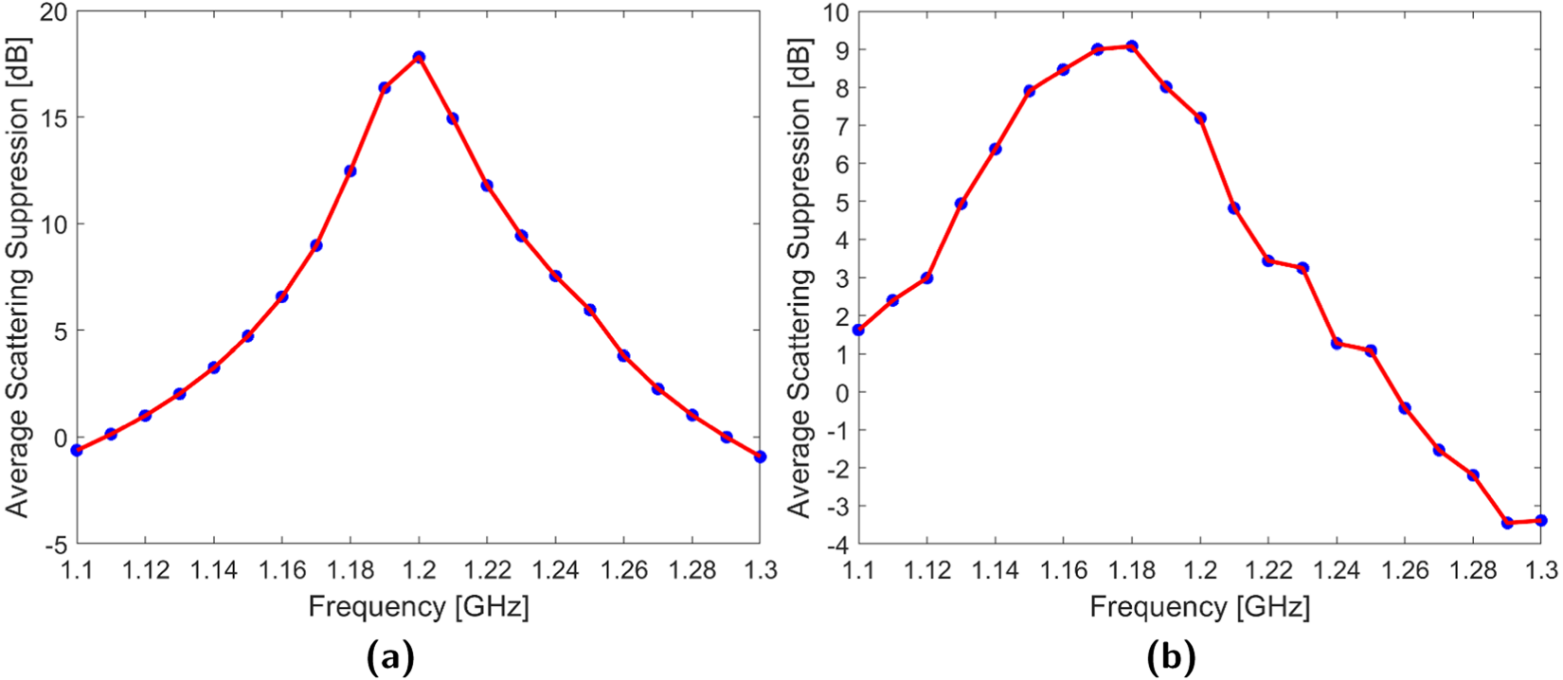
Single weight sweep at 1.2 GHz (**a**) Simulated (**b**) Experimental.

 Figure 9a plots the simulated average suppression achieved between 1.1 and 1.3 GHz when the cloak is configured for 1.2 GHz. It can be seen that the cloak possesses a 3 dB bandwidth of 10% and loses functionality outside 14%. Although the peak performance is lower and shifted to 1.18 GHz, the experimental cloak (Fig. 9b) possesses similar 3 dB and functional bandwidths of 9.2% and 13.3% respectively. It must be re-emphasized that multi-frequency behavior is dependent on cloak design and operating environment. For instance, the use of monopole elements results in a more narrowband response while controller capabilities may lead to frequency-dependent response variations. Operationally, if the incident wavefront varies in time and frequency consistently, it may be possible to design a single-weight cloak with a specific wideband response. Alternatively, assessment of single weight bandwidth is useful for determining the needed resolution for a multi-weight cloak and to assess the possibility of interference between overlapping excitations.

## Discussion

This work details the experimental demonstration of an active cloak designed to suppress the scattering of a non-uniform, metallic, polygonal target. In contrast to the uniform, circular cylinders used in previous experiments, the resultant scattering behavior becomes geometrically dependent on incident angle. Additionally, a polygonal target geometry allows evaluation of active cloaking performance when operating in the presence of flat surfaces and edges. The cloak itself is comprised of a thin, conformal network of commercially available monopoles surrounding the target. These are favored due to their reliable performance, low-profile, and robustness compared to previous element designs. The resultant array is then configured to cancel out scattered fields which naturally arise when the target is illuminated by an impinging electromagnetic wave. To impose quasi-2D conditions, the target/cloak assembly is enclosed within a parallel-plate waveguide where it is illuminated at either a frontal or an oblique incidence by a 1.2 GHz cylindrical wave.

Total field measurements for both incidence cases indicates that the cloak successfully suppressed scattering over the entire azimuth. An average suppression of 7.2 dB was recorded at the frontal, $\theta ^{\prime} ={0}^{\circ }$ incidence case while 8.6 dB mean suppression is achieved in the oblique, $\theta ^{\prime} =3{0}^{\circ }$ incidence case. Both local suppression minima and maxima were present at the edges of the target for both cases, suggesting that edge effects did not significantly impact cloak performance. Although not originally a design objective, experimental results combined with simulations were used to demonstrate that the cloak could be reconfigured for single frequency operation across 0.8–1.8 GHz. This “frequency hopping” evaluation revealed that performance may be degraded by additional resonance scattering near the antennas’ operating frequency as well as current phase inversion and array aliasing at higher frequencies. Lastly, it was found that the cloak possessed 3 dB and functional bandwidths of approximately 10% and 14% respectively when weighted for operation at 1.2 GHz. It is important to note that multi-frequency performance was only specific for this particular cloak design, operating environment, and incident wave.

Future development of this cloaking technique includes cloak designs that accommodate 3D targets and the development of systems to estimate the incident field. As most real-world objects are not 2D, the need for any practical cloak to occlude 3D targets is self-evident. However, such cloaks will need to contend with varying incident elevations and polarizations along with a 3D target’s increased size and complexity. Furthermore, all designs and experiments in this method have operated on a priori knowledge of the incidence field. Although useful for proof of concept purposes, a practical cloak is not likely to possess prior knowledge of the impinging wave and may also need to detect and adapt to signal changes. Aside from the development of a control system, an adaptive cloak will also require the ability to sense the total field within its vicinity and a means of deducing the scattered field from these measurements. Field sensing may be accomplished using a separate sensor array^24^ or the cloaking elements themselves^26^ while the total and scattered fields may be related through the target’s Green’s function^34^. Lastly, the discussion of multi-frequency operation provides possible developmental basis for a cloak capable of operating over a range of frequencies simultaneously. This can be accomplished with a weighting controller capable of adaptively enforcing weights at different frequencies simultaneously to custom tailor a frequency varying cloak field.

## Methods

### Simulations

Ansys HFSS, a full-wave electromagnetic, finite-element solver was used to model the cloak along with the experimental waveguide environment. The resultant simulation was first employed to aid in cloak and apparatus design, validate array configurations, and provide initial performance estimates. As experimentation progressed, simulated results were used to complement and extrapolate experimental results. This enabled an expanded analysis of the cloak’s multi-frequency capabilities in-spite of experimental apparatus limitations.

### Experimental apparatus

A two-port Agilent E8364 programmable network analyzer (PNA), acting as both the signal source and primary measurement device, forms the core of the cloak and experimental apparatus. Port 1 is designated as the transmit port and generates a signal at operating frequency. This is sent to the cloak’s weighting controller (Fig. 2b) and divided into 16 channels. Voltage biased attenuators (Mini-Circuits EVA-1500) and phase shifters (Mini-Circuits SPHSA-152) situated on each channel are then used to regulate the magnitude and phase of each element input. To allow the cloak to be weighted with respect to the source, 15 of these channels are linked to the cloak array monopoles while the remaining channel feeds a source monopole responsible for producing the incident wave. Biasing voltages for the channel controllers originate from a Kikusui PMC18-2A DC power supply and distributed via a computer controlled data acquisition system (Measurement Computing USB-3105). The individual operating ranges of the attenuators and phase shifters are 100–1500 MHz and 800–1500 MHz respectively. In practice, it was found that phase shifter range and overall weighting stability degrades at frequencies higher than 1.2 GHz.

Both the target and two waveguide plates are milled from aluminum stock and plate. The target measures: *W* = 218.18 mm, *L* = 272.73 mm, *ϕ* = 45°, and *h* = 38 mm (Fig. 1) while the plates are 1626 × 1094 mm. Holes are milled in the bottom plate to facilitate consistent mounting of the monopoles, via SMA bulkhead connectors, and the metallic target. Cloaking monopoles (Taoglas GW.26.0111) are mounted *w*/2 = 14 mm from the target’s surface, with three evenly spaced antennas per face, while inter-elemental spacing is maintained at less than half-wavelength to prevent array aliasing. The source monopole is then installed in one of two holes situated $\rho ^{\prime} =600$ mm from the target’s center, allowing the target to be illuminated from either $\theta ^{\prime} ={0}^{\circ }$ or $\theta ^{\prime} =3{0}^{\circ }$ incidence. To impose free-space conditions, the perimeter of the waveguide is lined with foam absorbers. Aluminum spacers situated outside the absorbers separate the top and bottom waveguide plates by *h* = 38 mm, equal to the height of the target. As this spacing is less than half-wavelength, non-TEM modes are suppressed and a quasi-2D environment is created. The top plate of the waveguide contains a milled 80 × 40 array of 10 mm diameter perforations with 15 mm spacing. This permits the fields within to be mapped by inserting a coaxial probe, connected to the receiving (Port 2) of the PNA, into each perforation and measuring *S*_21_. To automate the mapping process, the probe is affixed to a computer controlled translator stage which scans a 552.24 × 570.4 mm section.

## Supplementary information

Supplementary Information.

## References

[1] Schurig D (2006). Metamaterial electromagnetic cloak at microwave frequencies. Science.

[2] Kim Y, Seo I, Koh I-S, Lee Y (2016). Design method for broadband free-space electromagnetic cloak based on isotropic material for size reduction and enhanced invisibility. Opt. Express.

[3] Ma, Y. *et al*. First experimental demonstration of an isotropic electromagnetic cloak with strict conformal mapping. *Sci. Reports* **3** (2013). 10.1038/srep02182 PMC3711045 23851589

[4] Chen, H. & Zheng, B. Broadband polygonal invisibility cloak for visible light. *Sci. Reports* **2** (2012). 10.1038/srep00255 PMC3275922 22355767

[5] Rainwater D (2012). Experimental verification of three-dimensional plasmonic cloaking in free-space. New J. Phys..

[6] Vitiello, A. *et al*. Waveguide characterization of s-band microwave mantle cloaks for dielectric and conducting objects. *Sci. Reports* **6** (2016). 10.1038/srep19716 PMC4726170 26803985

[7] Qin, F. F., Liu, Z. Z., Zhang, Q., Zhang, H. & Xiao, J. J. Mantle cloaks based on the frequency selective metasurfaces designed by bayesian optimization. *Sci. Reports* **8** (2018). 10.1038/s41598-018-32167-x PMC6145948 30232342

[8] Zhou, F. *et al*. Hiding a realistic object using a broadband terahertz invisibility cloak. *Sci. Reports* **1** (2011). 10.1038/srep00078 PMC3216565 22355597

[9] Lan, C., Yang, Y., Geng, Z., Li, B. & Zhou, J. Electrostatic field invisibility cloak. *Sci. Reports* **5** (2015). 10.1038/srep16416 PMC4639767 26552343

[10] Farhat, M. *et al*. Thermal invisibility based on scattering cancellation and mantle cloaking. *Sci. Reports* **5** (2015). 10.1038/srep09876 PMC4415578 25928664

[11] Yang, Y., Wang, H., Yu, F., Xu, Z. & Chen, H. A metasurface carpet cloak for electromagnetic, acoustic and water waves. *Sci. Reports* **6** (2016). 10.1038/srep20219 PMC4731745 26822429

[12] Zhou, F. *et al*. Field-programmable silicon temporal cloak. *Nat. Commun*. **10** (2019). 10.1038/s41467-019-10521-5 PMC6586806 31222060

[13] Fleury R, Monticone F, Alù A (2015). Invisibility and cloaking: Origins, present, and future perspectives. and future perspectives. Phys. Rev. Appl..

[14] Monticone F, Alù A (2013). Do cloaked objects really scatter less?. Phys. Rev. X.

[15] Zeng, C., Liu, X. & Wang, G. Electrically tunable graphene plasmonic quasicrystal metasurfaces for transformation optics. *Sci. Reports* **4** (2014). 10.1038/srep05763 PMC4104397 25042132

[16] Hosseininejad, S. E. *et al*. Reprogrammable graphene-based metasurface mirror with adaptive focal point for thz imaging. *Sci. Reports* **9** (2019). 10.1038/s41598-019-39266-3 PMC6393480 30814570

[17] Meng, Q., Zhong, Z. & Zhang, B. Hybrid three-dimensional dual- and broadband optically tunable terahertz metamaterials. *Sci. Reports* **7** (2017). 10.1038/srep45708 PMC5372461 28358357

[18] Yang, H. *et al*. A programmable metasurface with dynamic polarization, scattering and focusing control. *Sci. Reports* **6** (2016). 10.1038/srep35692 PMC5075904 27774997

[19] Chen P-Y, Argyropoulos C, Alù A (2013). Broadening the cloaking bandwidth with non-Foster metasurfaces. Phys. Rev. Lett..

[20] Liu S, Xu H-X, Zhang HC, Cui TJ (2014). Tunable ultrathin mantle cloak via varactor-diode-loaded metasurface. Opt. Express.

[21] Farhat M, Rockstuhl C, Bağci H (2013). A 3d tunable and multi-frequency graphene plasmonic cloak. Opt. Express.

[22] Zhu X, Feng L, Zhang P, Yin X, Zhang X (2013). One-way invisible cloak using parity-time symmetric transformation optics. Opt. Lett..

[23] Zhu X, Ramezani H, Shi C, Zhu J, Zhang X (2014). $${\mathcal{P}}{\mathcal{T}}$$ -symmetric acoustics. Phys. Rev. X.

[24] Miller DAB (2006). On perfect cloaking. Opt. Express.

[25] Selvanayagam M, Eleftheriades GV (2012). An active electromagnetic cloak using the equivalence principle. IEEE Antennas and Wirel. Propag. Lett..

[26] Selvanayagam M, Eleftheriades GV (2013). Experimental demonstration of active electromagnetic cloaking. Phys. Rev. X.

[27] Ang, P. & Eleftheriades, G. V. Active Huygens’ cloaks for arbitrary metallic polygonal cylinders. In *2018 IEEE/MTT-S International Microwave Symposium - IMS*, 337–340 (2018).

[28] Ang, P. & Eleftheriades, G. V. Active surface cloaking with patch antennas. In *2018 IEEE International Symposium on Antennas and Propagation* (2018).

[29] Ang, P. & Eleftheriades, G. V. Experimental active cloaking of a metallic polygonal cylinder. In *2019 IEEE/MTT-S International Microwave Symposium - IMS* (2019).

[30] Harrington, R.* Time-Harmonic Electromagnetic Fields*. (Wiley-IEEE Press, 2001).

[31] Balanis, C.* Antenna Theory: Analysis and Design* (Wiley, 2012).

[32] Wong, A. M. H. & Eleftheriades, G. V. Active Huygens’ metasurfaces for RF waveform synthesis in a cavity. In *2016 18th Mediterranean Electrotechnical Conference (MELECON)*, 1–5 (2016).

[33] Brown J, Jull EV (1961). The prediction of aerial radiation patterns from near-field measurements. Proc. IEE - Part B: Electron. Commun. Eng..

[34] Friot E, Bordier C (2004). Real-time active suppression of scattered acoustic radiation. J. Sound Vib..

