# Supplementary material for: Active Cloaking of a Non-Uniform Scatterer
Source: Sci Rep. 2020 Feb 6;10:2021. doi: 10.1038/s41598-020-58706-z (PMC7005261; doi:10.1038/s41598-020-58706-z)
Supplement: Supplementary file 1 — Supplementary Information. [file 41598_2020_58706_MOESM1_ESM.pdf]

Supplementary Information: Active Cloaking of a Non-Uniform Scatterer

Paris Ang[1,*,X] **and George V. Eleftheriades**[1,+]

[1]University of Toronto, The Edward S. Rogers Sr. Department of Electrical and Computer Engineering, Toronto, M5S 2E4, Canada
[*]paris.ang@mail.utoronto.ca
[+]gelefth@waves.utoronto.ca
[X]Corresponding Author

ABSTRACT

This document presents supplementary information relevant to the main paper.

Additional incident angles

While only two incident angles were evaluated experimentally, the cloak is capable of being reconfigured to accommodate an impinging wave from any direction. To demonstrate this, the multi-frequency simulation model can be weighted to operate against illumination by a 1.2 GHz cylindrical wave at $\theta' = 150°$ and $\theta' = 180°$ incidence. As the target is symmetric, the total four incidence cases provide a comprehensive picture of the cloak's directional reconfigurability.

Fig. S1 plots the cloak OFF/ON normalized real E-field plots and the bistatic RCS patterns at $\theta' = 150°$ incidence. The resultant E-fields resemble a reversed $\theta' = 30°$ scenario where the wave impinges on the edge of the target rather than a flat surface. Furthermore, Fig. S1c indicates an area of increased suppression around $-100°$ azimuth; responsible for an elevated average scattering suppression of 19 dB. Of note, is that the elements in this location are inline with the wave's constant phase direction. This may allow them to more accurately approximate a cylindrical wavefront behind the target.

The $\theta' = 180°$ incidence case is similarly detailed in Fig. S2. Likewise, the pattern resembles an inverted $\theta' = 0°$ scenario with an average scattering suppression of 18.2 dB. As the incident wave directly impinges on a large flat surface, the cloak OFF (and ON) maximum resides at the illuminated face of the target (0°) rather than within the shadow region. Furthermore, the cloak OFF peak corresponding to the shadow region possesses a finer gradient and improved suppression possibly due to the ramping of the rear facing surfaces.

To provide direct comparison, Fig. S3 and Fig. S4 detail the results of simulations at $\theta' = 0°$ and $\theta' = 30°$ incidence. These indicate an average scattering suppression of 17.8 dB and 18 dB respectively, showing that theoretical performance is largely incident angle independent.

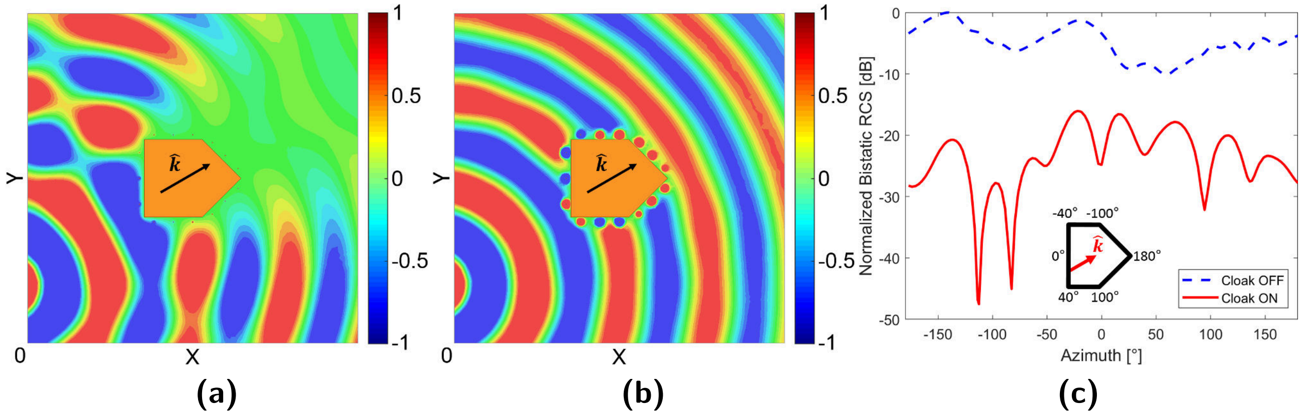

Figure S1. $\theta' = 150°$ incidence (a) E-field OFF (b) E-field ON (c) Bistatic RCS

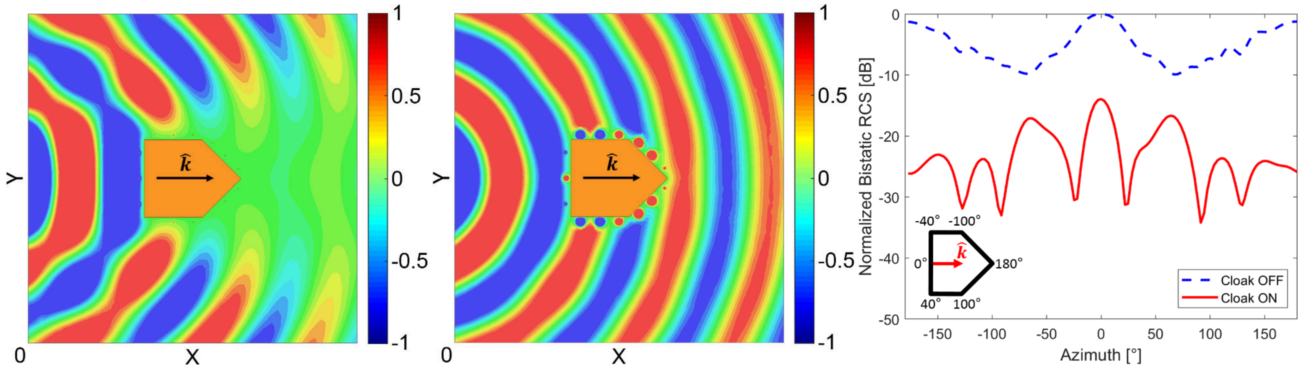

(a) (b) (c)

Figure S2. $\theta' = 180^{\circ}$ incidence (a) E-field OFF (b) E-field ON (c) Bistatic RCS

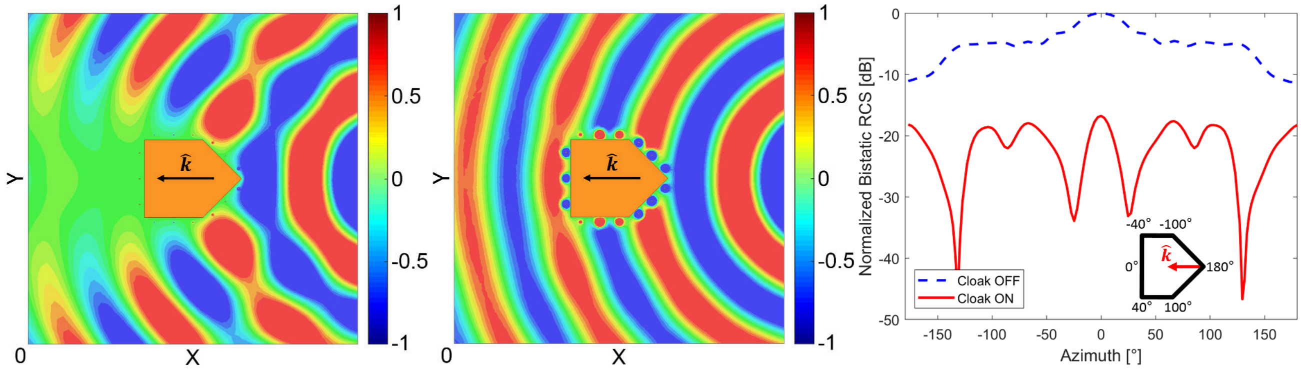

(a) (b) (c)

Figure S3. $\theta' = 0^{\circ}$ incidence (a) E-field OFF (b) E-field ON (c) Bistatic RCS

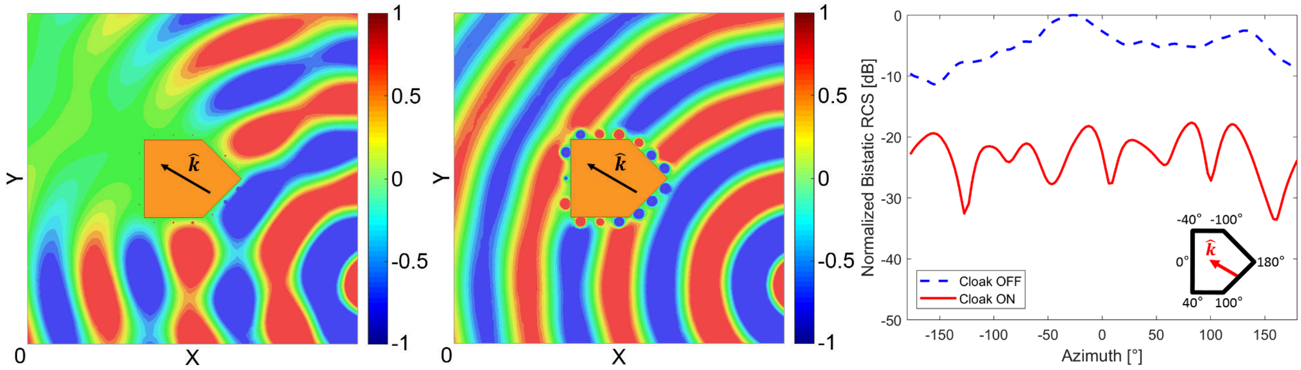

(a) (b) (c)

Figure S4. $\theta' = 30^{\circ}$ incidence (a) E-field OFF (b) E-field ON (c) Bistatic RCS

Complex incident wavefronts

To simplify apparatus design and provide detailed insight into the weighting process, the cloak experiment utilizes a monopole antenna (of the same make as those comprising the cloak) to generate the incident wave. The resultant incident cylindrical wavefront can then be analytically characterized, allowing the required element weights to be easily determined. However, a drawback of this weighting method is that it requires detailed knowledge of the wavefront geometry, source location, and the source's input amplitude and phase. This becomes impractical for complex waveforms and in situations where source information is not available. As the cloak only requires knowledge of the scattered field at the target's surface for functionality,

it is possible to perform element weighting with local information. One means of doing so is to take localized measurements of the incident field, within an empty waveguide, at the would-be locations of each cloak element. As these are located near the target's surface, the scattered field is the negative of the incident field, allowing the appropriate weights to be calculated directly. This configuration method is demonstrated in the following two cases which feature the introduction of a second radiating source to generate a complex incident wavefront.

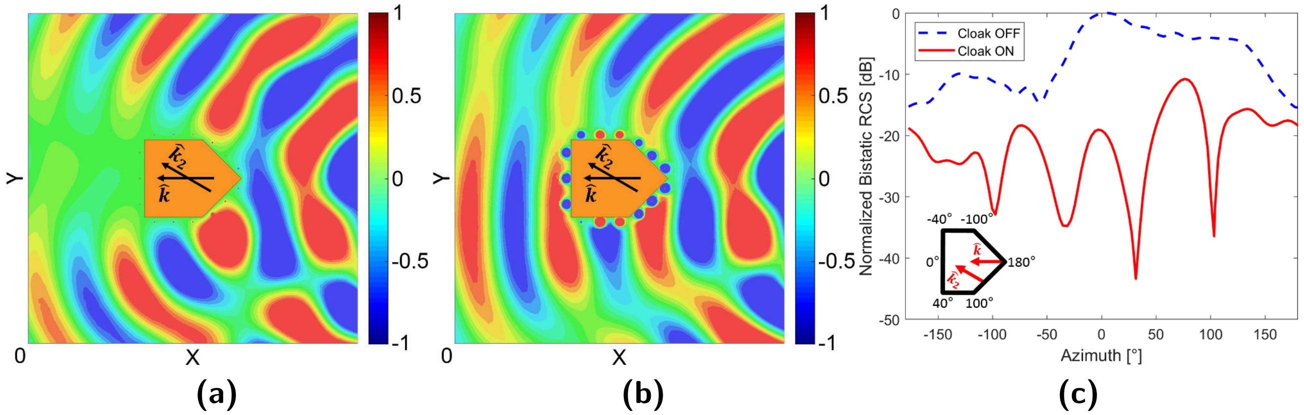

Figure S5. Case 1 (a) E-field OFF (b) E-field ON (c) Bistatic RCS

The first scenario, Case 1, illustrated in Fig. S5 adds a second source antenna at $\theta_2' = 30°$ incidence to a $\theta' = 0°$ incidence case. To further complicate the problem, the second source's input phase is shifted by $-90°$ and its amplitude attenuated by 3 dB; with respect to the amplitude of the initial $\theta' = 0°$ incidence source. Although the resultant wavefront is still roughly cylindrical, interference between the sources creates two radially extending nulls. The effect of one of these nulls manifests as reduced cloak OFF scattering (Fig. S5c) between $-180°$ and $-50°$ azimuth, corresponding to where it intersects the target. Although the field plots indicate that the cloak is successful at reconstructing the incident field, the average suppression is slightly reduced to 14.3 dB. This is likely due to the cloak's propensity of having reduced suppression at the location of naturally low scatterers; suppression minima were found to occur within areas with low cloak OFF scattering, such as the incident null region or near the directly illuminated corners at 180° and 100° azimuth.

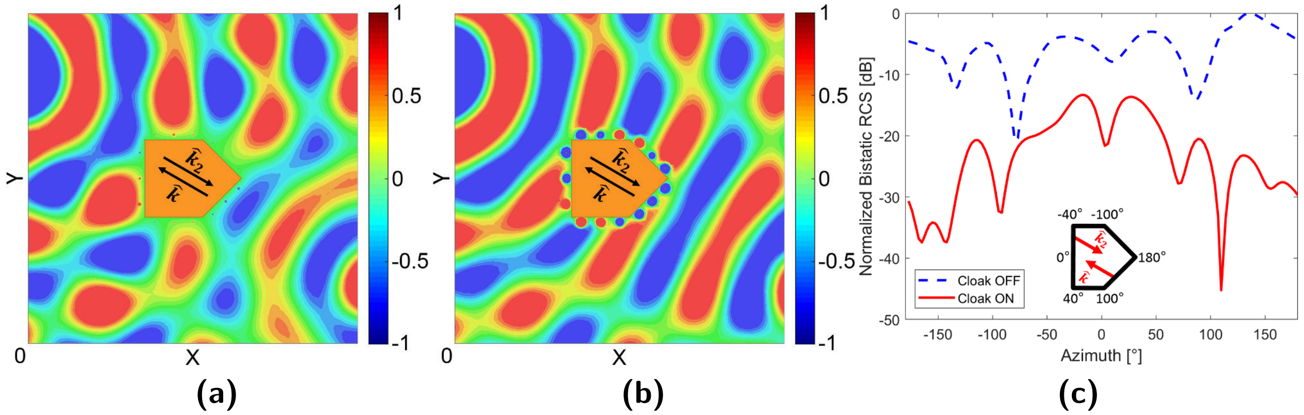

Figure S6. Case 2 (a) E-field OFF (b) E-field ON (c) Bistatic RCS

Case 2 in Fig. S6 features the target in between the two incident sources. Here, the first source, with a 20° phase shift and 3 dB attenuated amplitude, is placed at an incident angle of $\theta' = 30°$. The second source antenna, phase shifted by $-90°$ and with no amplitude reduction, is positioned at $\theta_2' = -150°$ incidence. This results in the target being enclosed within a planar wavefront. The resultant average suppression of 17.6 dB is more comparable to single source simulated cases while overall suppression is achieved across the entire azimuth. A suppression minima is observed near $-75°$ azimuth which may be associated with the low natural scattering of the $-40°$ corner. It should be noted that in both cases, the sources were kept at the same radial distance ($\rho' = \rho_2' = 600$ mm) to keep far-field and RCS calculations simple. Comparison of near-field scattering in previous simulations verify functionality in both cases when the second source's distance is reduced to $\rho_2' = 500$ mm.

The direct calculation of cloak weights from surface incident field measurements demonstrates that the cloak is capable of operation in scenarios where detailed, a priori knowledge of the incidence source(s) is unavailable or the incident field is too complex for practical analysis. Although more capable, this method is still demanding from a practical perspective as it may not always be possible or practical to take incident field measurements. Subsequently, a pertinent research focus is the development of a means to determine the scattered field directly from total field measurements, such as by means of the object's Green's function, allowing measurements to be taken within the target's vicinity. Such a formulation would also ease the development and implementation of adaptive sensing and control capabilities.
